# Feminist or Paternalistic: Understanding Men’s Motivations to Confront Sexism

**DOI:** 10.3389/fpsyg.2019.02988

**Published:** 2020-01-17

**Authors:** Lucía Estevan-Reina, Soledad de Lemus, Jesús L. Megías

**Affiliations:** Mind, Brain and Behaviour Research Centre, University of Granada, Granada, Spain

**Keywords:** feminist identification, benevolent sexism, egalitarian motivation, paternalistic motivation, sexism confrontation, collective actions, allies, social change

## Abstract

The role of men in fighting gender inequality is a controversial issue. Literature has shown that advantaged group members can promote social change but also perpetuate *status quo*. We conducted three studies to examine two motivational processes that may lead men to confront sexism: an egalitarian path and a paternalistic one. Studies 1–3 revealed that men high in benevolent sexism were more willing to confront sexism for paternalistic reasons, whereas Studies 2–3 found that men high in feminist identification were more likely to confront sexism for egalitarian reasons. Pooled analyses (Studies 1–3) supported the egalitarian and paternalistic paths underlying sexism confrontation. Moreover, Studies 2 and 3 extended these findings to collective action and engagement in the men’s activist movement that aims to reflect on male privilege (i.e., the Men for Equity movement). These results highlight the existence of various underlying motivations to confront sexism by men, as well as the limits of paternalism and the potential of feminism to motivate men to take part in other kinds of actions beyond confrontation to foster social change.

## Introduction

The fight against gender inequality has traditionally been seen exclusively as “a women’s issue” due to the fact that women are the targets of discrimination. Thus, it has been assumed that they are the ones concerned with improving women’s positions in society (Tajfel and Turner, 1979; Wright et al., 1990). However, the literature reflecting upon political solidarity and the role of advantaged group members as allies in promoting social change has gained wide attention in the last decade (Subašić et al., 2008; McGarty et al., 2009; van Zomeren et al., 2011). Scholars have recently studied the particular role of men in fighting gender inequality (Good et al., 2016; Radke et al., 2018). The aim of this paper is to extend these findings by examining the underlying motivational processes that lead men to confront sexism.

Members of privileged groups can be more effective in confronting prejudice compared to disadvantaged group members because they experience fewer costs and more benefits of confrontation (Kaiser and Miller, 2001). For instance, it is less probable that they are perceived as complainers (Gulker et al., 2013). However, whether confrontation by advantaged groups implies real social change remains an important research question. In fact, high-status groups’ helping behavior may contribute in some cases to legitimizing the privileged status at the same time that the disadvantaged group feels grateful and legitimizes social inequality (Nadler, 2002). However, advantaged group members can also become allies and promote real social change (Subašić et al., 2018), but it seems that some conditions must be fulfilled for this to happen. For instance, Becker et al. (2013) found that positive contact between advantaged and disadvantaged groups may undermine collective action among the disadvantaged unless the advantaged group has made explicitly clear that the inequality being experienced is illegitimate. According to Drury and Kaiser (2014), for men to be deemed allies of women against sexism, they must work *alongside* rather than *on behalf of* women. The identification of ideological and motivational factors underlying confrontation of sexism may contribute to understanding when men are working alongside or on behalf of women. In this work, we aim to study how these factors behave in a situation where men confront a sexist comment. We specifically argue that men confronting sexism may be motivated by feminist reasons but also by paternalistic ones. Motivations mediate the relationship between attitudes and behaviors (Higgins and Kruglanski, 2000). We argue that endorsing egalitarian goals can lead men to confront sexism as a strategy to promote social change. However, paternalistic goals can also lead men to confront sexism. Good et al. (2016) found that the duty to protect women may be one of the motives that lead men to confront sexism, thus paradoxically reinforcing the *status quo*. Understanding the motivational processes underlying male confrontation of sexism is essential for determining when such confrontation may help or hinder gender inequality. Thus, the main purpose of this work is to test the existence of these two motivational processes—confrontation based on feminist versus paternalistic reasons—which could have very different implications for stability and social change.

### The Role of Feminist Identification and Egalitarianism in Promoting Social Change

Men may promote social change by confronting sexism if they are actively committed to fighting gender inequality. In fact, the more men endorse feminist beliefs, the more aware of sexism they are (Swim et al., 2001), the more they reject modern sexist beliefs (Swim et al., 2004), and, in turn, the more incidents of sexism they report (Swim et al., 2004; Hyers, 2007). Wiley et al. (2012) found that positive portrayals of feminist men increased men’s sense of solidarity with feminists, which leads to increasing their intentions to engage in collective action. Subašić et al. (2018) similarly found that considering men as agents of change toward gender equality increases men’s collective action intentions, as well as feminist solidarity and the perceived illegitimacy of gender inequality. In addition, researchers recently showed a positive relationship between men’s feminist identification and their willingness to take part in feminist actions (Radke et al., 2018), but evidence of the role of feminist identification in predicting sexism confrontation is still lacking. In the case of women, literature has shown that although holding feminist attitudes does not mean embracing the feminist label (Zucker, 2004; Zucker and Bay-Cheng, 2010), embracing the feminist label usually means having egalitarian attitudes (Duncan, 2010) and is positively related to confronting sexism (Weis et al., 2018). Ellemers et al. (2002) suggested the importance of differentiating between identification (the strength of people’s ties with a particular group) and social identity (nature or content of a particular identity). In this way, egalitarian attitudes, values, beliefs, and motivations should be part of the content of the feminist identity, and feminist identification should imply the strength with which a person embraces the label associated with such an identity. Therefore, we argue that the strength of feminist identification in men will predict their confrontation intentions (and other actions more related to social change) because it activates egalitarian motivations in men. We defined egalitarian motivation as the force that leads men to act in order to achieve the following goals: raising awareness of gender inequality (Swim et al., 2001; Drury and Kaiser, 2014), acknowledging that some attitudes and beliefs perpetuate and legitimize inequality (Gurin, 1985; Drury and Kaiser, 2014), promoting social change orientation [close to the collective orientation component of gender awareness that Gurin (1985) proposed], and showing men’s engagement in fighting gender inequality (Subašić et al., 2008; Drury and Kaiser, 2014). Thus, we hypothesized that egalitarian motivation will mediate the relationship between feminist identification and sexism confrontation.

### The Role of Benevolent Sexism and Paternalism in Perpetuating Inequality

The literature has confirmed the idea that to maintain inequality, benevolence is more effective than hostility (Jackman, 1994). Glick and Fiske (1996) proposed that two coexisting forms of sexism are positively related and work together: hostile sexism (related to the traditional way of understanding prejudice as negative attitudes toward women) and benevolent sexism (prejudice expressed with a positive tone, with the assumption that women must be cherished and protected by men). Barreto and Ellemers (2005) revealed that the positive tone of benevolent sexism makes it difficult to recognize as a form of prejudice and might also have some apparently positive effects. For instance, Shnabel et al. (2016) and Radke et al. (2018) found that benevolent sexism may play an important role in promoting cross-gender helping relations and in promoting collective action intentions among men on behalf of women. However, these authors warned that benevolent sexism promotes dependency-oriented cross-gender helping relations but not autonomy-oriented ones (Shnabel et al., 2016) and protective collective actions but not feminist collective actions (Radke et al., 2018). Therefore, benevolent sexism may ironically promote some action against inequality while reinforcing sexist views of women as unable to stand up for themselves.

In fact, Glick and Fiske (1996) differentiated between *dominative paternalism* based on the idea that women are not fully competent adults and *protective paternalism*, which is based on men’s dyadic dependence on women as wives, mothers, and romantic objects, who should be loved, cherished, and protected by men. Good et al. (2016) found that this masculine protector role predicted confronting sexism, although only toward socially close women (e.g., a girlfriend, mother, or sister). However, we consider that it is important to disentangle the role of benevolent sexist beliefs and paternalistic motivations as predictors of sexism confrontation in general. Sexist beliefs are a more stable construct (Huang et al., 2019) that may trigger specific paternalistic motivation in response to a given sexist situation and lead to confrontation responses. Whereas Good et al. (2016) emphasized the link between paternalism and confrontation, Radke et al. (2018) focused on the link between benevolent sexism and protective action. We propose an overall conjoint model that considers both the role of sexist attitudes and paternalistic motivations sequentially in predicting sexism confrontation. We hypothesized that paternalistic motivation specifically mediates the relationship between benevolent sexism and sexism confrontation. However, because the underlying principle of paternalism is that women are dependent on men, we expect that paternalistic motivations will not be related to actions with higher implications for social change (e.g., supporting feminist actions and engagement in groups that question male privileges).

### Current Studies

In this work, we are interested in disentangling two motivational processes that might lead men to confront sexism. In doing so, it will be possible to understand the conditions in which men can be true allies of women in fighting against gender inequality (Drury and Kaiser, 2014). In Studies 1–3, we examine the role of sexism and feminism as ideological predictors that activate egalitarian versus paternalistic motivations and, in turn, trigger sexism confrontation in men. Furthermore, we want to analyze the boundaries of paternalism in promoting social change compared to feminism. To achieve this goal in Studies 2 and 3, we examine whether the motivational processes proposed could be extended to understanding other responses of men against inequality (as collective action intentions in favor of women’s rights and engagement in the Men for Equity movement). We expect that feminist identification and egalitarian motivation will also predict these types of actions, whereas benevolent sexism and paternalistic motivation will not. Finally, we conducted an Integrative Data Analysis with the three data sets pooled into one (Curran and Hussong, 2009) to increase statistical power and sample heterogeneity.

## Study 1

The goal of Study 1 was to uncover the two motivational processes leading men to confront sexism. As explained previously, we expected the relation between feminist identification and sexism confrontation to be mediated by egalitarian motivation (Hypothesis 1). Furthermore, we expected the relation between benevolent sexism and confronting sexism to be mediated by paternalistic motivation (Hypothesis 2).

### Method

#### Participants

A total of 150 men completed the study. We detected four univariate outliers in the amount of time that they spent answering the survey. After we checked the cases, we decided to delete them because they spent more than 1 day with the survey open. Two multivariate outliers were detected, so we also excluded them. The final sample was 144 men. Participants’ ages ranged from 18 to 63 years, with a mean of 22.32 years (*SD* = 5.87). All of them were students from a university in southern Spain, and 96.5% were Spanish citizens. We conducted a sensitivity analysis using G^∗^power (Faul et al., 2007) to determine the effect size the current study could detect. The results showed that with this sample size (*N* = 144), with α = 0.05 and 1-*β* = 0.80, the minimum effect size that we can detect for a multiple regression analysis with two predictors is *f* ^2^ = 0.07.

#### Measures

Unless differently specified, participants were asked to rate each measure on a scale from 1 (*strongly disagree*) to 7 (*strongly agree*). The measures are described here in the same order as they appeared in the online survey. For exploratory purposes, we included a number of additional variables that are not described in the main text but have been listed and explained in more detail at https://osf.io/tdfmb/.

##### Demographics

We recorded participants’ ages, nationality, and academic major.

##### Gender and feminist identification

We measured gender and feminist identification with the centrality and solidarity items of Leach et al.’s (2008) scale adapted to gender and identification with feminist people. Seven items made up the measure; three of them captured the idea of centrality (e.g., “The fact that I am a [man/feminist person] is an important part of my identity”), and three more captured the idea of solidarity (e.g., “I feel a bond with [men/feminist people]”). In addition, we included a general item in which we asked participants the extent to which they perceived themselves as feminists (Doosje et al., 1998; men: α = 0.97; feminist people: α = 0.95).

##### Hostile and benevolent sexism

We measured hostile and benevolent sexism using the Spanish version of Glick and Fiske’s (1996) Ambivalent Sexism Inventory (Expósito et al., 1998). The scale included 11 hostile sexism items (e.g., “Most women interpret innocent remarks or acts as being sexist”; α = 0.93) and 11 benevolent sexism items (e.g., “Many women have a quality of purity that few men possess”; α = 0.85) answered from 0 (*strongly disagree*) to 5 (*strongly agree*).

##### Sexist situations

Based on the descriptions that other university students (N = 19) provided in a qualitative pilot study and to increase the ecological validity of our research, we created three scenarios representing sexist situations that young women may suffer. One scenario was an episode of street harassment in which a young woman passes by a dark street and a man says: “Hi beautiful, where are you off to so alone? Why don’t you hang out with me for a while?” Another scenario presented an insinuation to provide help in exchange for some kind of sexual contact—quid pro quo—in a bus station when a young woman asks the guard whether he can keep her luggage for a while and he answers: “If you behave well with me, I can also behave well with you.” The third scenario involved a sexist comment at the entrance to a pub when a young woman asks some men for a lighter and one of them responds: “I will lend it to you, pretty girl, but if in return you come to sleep with me because tonight I don’t want to sleep alone.” Each participant received one of these randomly assigned scenarios (more information about scenarios may be found in https://osf.io/tdfmb/). Even when differences existed in how sexist, threatening, and insulting the scenarios were perceived to be, we found no significant effect of the type of scenario on the main dependent variables (paternalistic motivation, egalitarian motivation, and confrontation), therefore results were averaged across scenarios for the purpose of the analyses.

##### Intentions of assertive sexism confrontation

We measured intentions of assertive sexism confrontation with a pool of items based on previous literature (Swim and Hyers, 1999; Hyers, 2007; Becker et al., 2015). After reading the sexist scenario, we asked participants to what extent they would react in different ways to the sexist comment. Following the distinction that Hyers (2007) proposed, we included five items to capture the goal of educating the perpetrator (e.g., “I would make him reflect upon his comment”) and three items to capture the goal of self-validation (e.g., “I would tell him that I cannot keep silent in front of his comment”). In addition, we included five items to explicitly manifest the disagreement with the comment (e.g., “I would tell him that I disagree with his comment”). A factorial analysis conducted following the principal component method of extraction with oblimin rotation revealed a unidimensional structure, so we decided to unify all of these items in one scale that we labeled assertive confrontation according to the previous literature (Hyers, 2007). These 13 items comprised a reliable scale (α = 0.96) and were answered from 1 (sure I would not act like that) to 7 (sure I would act like that).

##### Motivations underlying assertive sexism confrontation

These motivations were evaluated with a pool of items generated ad hoc for this study. We asked participants for the motives that would drive them to confront sexism in a situation such as that described in the scenario. Seven items assessed paternalistic motivations (e.g., “to show that a good man must protect women”) and eight items assessed egalitarian motivations (e.g., “to try to end the discrimination women suffer in their daily lives”). Our motivational items were specifically designed to tap into the underlying motivations driving behavior (confrontation of sexism) in that situation. To make sure that paternalistic motivation is clearly distinguishable from benevolent sexism, we conducted a factorial analysis including both paternalistic motivation and benevolent sexism items. Principal components analysis with varimax rotation (to avoid the factors to covary) extracted four factors with eigenvalues larger than one explaining 66.55% of variance. Paternalistic motivation items loaded on a separate factor (loadings 0.87–0.63), including only one item of protective paternalism that loaded in both factors; the rest of the items were distributed among the three factors reproducing the tridimensional structure of benevolent sexism, namely: heterosexual intimacy, complementary gender differentiation and protective paternalism (Glick and Fiske, 1996). Furthermore, we conducted another factorial analysis to confirm the distinction between paternalistic and egalitarian motivations. Principal components factor analysis with oblimin rotation extracted two factors with eigenvalues larger than one explaining 62.09% of variance. Paternalistic motivation (loadings 0.89–0.67) and egalitarian motivation items (loadings 0.83–0.69) loaded on the separate factors. We decided to exclude three items from further analyses because they showed high factorial loadings in both dimensions. The final set of items formed two reliable scales for measuring both paternalistic motivation (four items; α = 0.83) and egalitarian motivation (eight items: α = 0.90).

#### Procedure

We collected data online. Staff members distributed among their students the link to a survey (designed through the Qualtrics Platform) and encouraged them to participate in the research. In some cases, participants received extra credit courses as reward for their participation; when this was not possible, we offered them participation in a raffle for 30 euros. It took participants an average of 30 min to complete the study. At the end, we thanked the participants for their collaboration.

### Results

#### Analytical Strategy

We conducted correlational analyses to test the relationship between our variables (coefficients are shown in Table 1). Then we did mediation analyses with PROCESS (Preacher and Kelley, 2011) to test the role of motivations as potential mediators of the relationships between feminist identification and benevolent sexism with assertive sexism confrontation. We used 5,000 bootstrap samples to estimate bias-corrected standard errors and 95% percentile confidence intervals for the indirect effects. To control for the possible effect of the different scenarios, we included them as covariates in the mediation analyses.

**TABLE 1:** Means, standard deviations, and correlations for Study 1.

	**Mean (*SD*)**	**1**	**2**	**3**	**4**	**5**	**6**
(1) Feminist Id.	3.99 (1.77)	–	–0.42^∗∗^	–0.24^∗∗^	0.50^∗∗^	–0.23^∗∗^	0.20^∗^
(2) HS	1.58 (1.13)		–	0.58^∗∗^	–0.47^∗∗^	0.48^∗∗^	–0.02
(3) BS	1.34 (0.92)			–	–0.14	0.60^∗∗^	0.05
(4) Egalitarian Mot.	5.57 (1.16)				–	0.02	0.22^∗∗^
(5) Paternalistic Mot.	3.72 (1.50)					–	0.17^∗^
(6) Assertive Confr.	3.78 (1.71)						–

*Id., Identification; HS, Hostile Sexism; BS, Benevolent Sexism; MRNS, Masculine Role. Norm Scale; Mot., Motivation; Confr., Confrontation; ^∗^*p* < 0.05, ^∗∗^*p* < 0.01.*

#### Feminist Identification Predicts Assertive Sexism Confrontation Through Egalitarian Motivation

**FIGURE 1:**
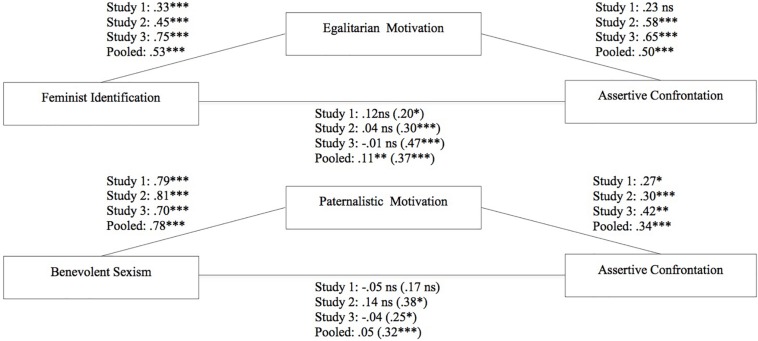
Motivational processes underlying men’s future intensions to confront Studies 1–3 and pooled analysis. Feminist path above and paternalistic path below. ^∗^*p* < 0.05, ^∗∗^*p* < 0.01, ^∗∗∗^*p* < 0.001.

We conducted a mediation analysis to test whether men’s feminist identification predicts sexism confrontation intentions via egalitarian motivation (Hypothesis 1). The total effect of feminist identification on sexism confrontation was significant (*b* = 0.20, 95% CI [0.04,0.35]). However, the direct effect was not significant when the egalitarian motivation was included as mediator (*b* = 0.12, 95% CI [−0.06,0.30]). The indirect effect via egalitarian motivation was also non-significant (*b* = 0.07, 95% CI [−0.01,0.19]) (see Figure 1 and Table 4). So Hypothesis 1 was not supported.

#### Benevolent Sexism Predicts Assertive Sexism Confrontation Through Paternalistic Motivation

Using the same analysis strategy, we examined whether the benevolent sexism of men predicts sexism confrontation intentions via paternalistic motivation (Hypothesis 2). As expected, benevolent and hostile sexism were correlated (*r* = 0.58, *p* < 0.01; Glick and Fiske, 1996; Glick et al., 2000). Hence, we included hostile sexism as a covariate to be sure that the potential variance shared between benevolent sexism and hostile sexism did not account for the results. In the mediation analysis, the total effect was not significant (*b* = 0.17, 95% CI [−0.21,0.55]). However, as hypothesized the indirect effect via paternalistic motivation was significant (*b* = 0.22, 95% CI [0.01,0.47]). The direct effect of benevolent sexism on assertive sexism was non-significant when we included the mediator (*b* = −0.05, 95% CI [−0.46,0.37]; see Figure 1 and Table 4). These results suggest that the effect of benevolent sexism on assertive sexism confrontation is mediated by paternalistic motivation. Thus Hypothesis 2 was supported.

### Discussion

These results did not support the existence of the feminist path (Hypothesis 1), because we found neither a direct effect of feminist identification on men’s intentions to confront nor indirect effect via egalitarian motivation. However, the data supported the hypothesis that paternalistic motivation mediates the relationship between benevolent sexism and assertive confrontation intention (Hypothesis 2), although the total effect was not significant. Thus, this study provides preliminary evidence in favor of the paternalistic path underlying sexism confrontation by men but not in favor of the feminist path. The absence of significant results favoring the hypothesized feminist path may be related to two factors, one theoretical and another methodological: (a) this process might play a more relevant role in explaining other types of actions against inequality than confrontation, for instance, collective actions; (b) according to the Monte Carlo simulation approach to estimating statistical power in mediation models, based in the correlations among the variables, the statistical powers for both paths with this sample size were below 0.60. For these reasons we decided to conduct a second study, first to explore the possible differences between paternalism and feminism in predicting other actions against gender inequality, such as men’s support for collective action for women’s rights (a closer psychological proxy for social change; van Zomeren et al., 2008) and men’s engagement in an activist movement aimed at reflecting on male privilege. The second purpose for Study 2 was to use a larger sample to increase the statistical power for these mediation analyses. In addition, we tried to increase the salience of feminist versus benevolent beliefs using an experimental manipulation.

## Study 2

In Study 2, we expected to find the two proposed paths underlying sexism confrontation among men with a larger sample, and we examined some potentialities of feminism and some boundaries of paternalism in promoting social change. To achieve our first aim, we hypothesized as in Study 1 that feminist identification predicts assertive confrontation through egalitarian motivation (Hypothesis 1a), whereas benevolent sexism predicts assertive confrontation through paternalistic motivation (Hypothesis 1b). Furthermore, we wanted to examine whether the motivational processes proposed may be extended so as to understand other kinds of responses against inequality, such as collective action in favor of women’s rights and engagement in the Men for Equity activist movement. Men for Equity is a male profeminist movement that started in the 1970s in the Nordic countries and has spread to other countries. The first groups in Spain appeared in the late 1980s (more information can be found at https://ahige.org/). We expected that the feminist path should play an important role in predicting collective actions supporting women’s rights, as well as interest in the Men for Equity movement, because these behaviors are directed to questioning the system and promoting social change (Radke et al., 2018). However, benevolent sexism and paternalism help to maintain the *status quo* and reinforce social hierarchies (Jost and Kay, 2005; Moya et al., 2007; Becker and Wright, 2011; Good et al., 2016; Shnabel et al., 2016; Radke et al., 2018). Thus, we hypothesized that the path based on feminist identification and egalitarian motivation may predict both support for collective action (Hypothesis 2a) and interest in the Men for Equity movement (Hypothesis 3a), whereas the path based on benevolent sexism and paternalistic motivation is not related to these outcomes (Hypotheses 2b and 3b). Finally, we analyzed whether these motivational processes predicted not only interest in but also actual engagement in the Men for Equity movement. We expected that the feminist path (Hypothesis 4a) but not the paternalistic one (Hypothesis 4b) predicts it.

### Method

#### Participants

A total of 204 men completed the survey. Participants’ ages ranged from 17 to 46 years old with a mean of 21.86 years (*SD* = 4.51). A participant was excluded because he was younger than 17 and we did not have approval from his parents to take part in the study. Using the same criteria as in Study 1, we checked for the presence of outliers. We detected three univariate outliers and excluded them based on the survey completion time. We also detected four multivariate outliers and excluded them. The final sample comprised 196 men. They were students from a university in southern Spain, and 96.9% were Spanish citizens. We conducted a sensitivity analysis to determine the effect size that the current study could detect. The results showed that with this sample size (*N* = 196), with α = 0.05 and 1-*β* = 0.80, the minimum effect size that we could detect for a multiple regression analysis with two predictors was *f* ^2^ = 0.05.

#### Measures

Unless something different is stated, participants were asked to rate each measure on a scale from 1 (*strongly disagree*) to 7 (*strongly agree*). The measures are described here in the same order as they appeared in the survey. We used the same measures as in Study 1 unless otherwise stated. In order to try to test experimentally our model we introduced a manipulation to increase the salience of feminist versus benevolent beliefs in men and two scenarios perceived as high or low in severity (based on the results from Study 1). However, neither manipulations produced significant effects in our dependent variables, therefore we did not described them in the main text and did not take them into account in the analyses (more information about manipulations and other extra variables can be found at https://osf.io/hvebt/). However, to control for the possible effect of these manipulations we included them as covariates in the mediation analyses.

##### Demographics

We recorded the ages, nationality, and the college career areas of participants.

##### Sexist situations

We randomly assigned participants to one of two scenarios (both used in Study 1: an episode of street harassment—high severity—or a sexist comment at the entrance of a bar—low severity).

##### Intentions of assertive confrontation

We used the same scale as in Study 1, but in this case with 12 items (α = 0.95; due to a technical error we missed data from one of the items).

##### Motivations underlying assertive sexist confrontation

The set of items formed a reliable scale for measuring both paternalistic (four items; α = 0.81) and egalitarian motivations (eight items; α = 0.91).

##### Collective action intentions

We included six items to measure men’s intentions to engage in collective action against gender inequality (e.g., “taking part in a strike for equal pay between women and men”; α = 0.79). Responses were recorded on a Likert scale from 1 (*surely I would not support that action*) to 7 (*surely I would support that action*).

##### Interest in the men for equity movement

We told participants about the existence in Spain of a Men for Equity movement and asked them if they were interested in knowing more about this movement. Responses were recorded using a Likert scale from 0 (I am not interested) to 10 (I am interested).

##### Intentions to engage in men for equity groups

As stated in the literature, a significant gap between intentions and actions exists (Sheeran and Webb, 2016). Therefore, we included two more questions that offered participants opportunities to actively engage in this activist movement. The first one asked participants if they wanted to receive more information about the Men for Equity groups in Spain. We explicitly stated that if they selected “yes,” we would provide them with brief information about the origin of Men for Equity groups, as well as the aims of this movement (an English translation version can be found at https://osf.io/hvebt/). However, if they selected “no,” then they would move on to the next section of the survey. Participants who selected “yes” were codified as 1, and participants who selected “no” were codified as 0. Those who selected “yes” read an extract from the website of the Association of Men for Gender Equity, which provided the history and goals of this movement in Spain (an English translation version can be found at https://osf.io/hvebt/). Then, we offered them the possibility of getting in touch with a local group of this movement and asked them to provide their e-mail addresses (second question). Participants who wrote their e-mail addresses were codified as 1, and those who did not were codified as 0. The results showed that 51.8% of the participants were open to receiving more information about the groups in Spain, but only 22.5% of them left their e-mail addresses (11.7% of the total participants). With the information obtained in both items, we created a composite variable ranging from 0 (*no engagement in these groups*) to 2 (*maximum engagement in these groups*).

##### Hostile and benevolent sexism

Hostile and benevolent sexism were measured with the Ambivalent Sexism Inventory (Glick and Fiske, 1996; Expósito et al., 1998) (SHα = 0.94; SBα = 0.87).

##### Feminist identification

We used the same seven items from Study 1 (α = 0.95).

#### Procedure

Participants were randomly assigned to either the feminist or benevolence salience condition and to one of the two sexist scenarios used (high vs. low severity). They first read a text making salient a feminist or benevolent norm (available at https://osf.io/hvebt/), followed by the scenario and a questionnaire including the measures of interest.

### Results

#### Analytical Strategy

We conducted correlational analyses to test the relationship between our variables (see Table 2). Then we ran mediational analyses with PROCESS macro to test the two paths leading to assertive confrontation as conducted in Study 1 (Hypotheses 1a and 1b), as well as to examine if the motivational processes proposed predicted men’s support for collective action (Hypotheses 2a and 2b), interest in the Men for Equity movement (Hypotheses 3a and 3b), and engagement in Men for Equity groups (Hypotheses 4a and 4b). Because the experimental manipulations did not have an effect on the dependent variables, we included them as covariates in all the analyses.

**TABLE 2:** Means, standard deviations, and correlations for Study 2.

	**Mean (*SD*)**	**1**	**2**	**3**	**4**	**5**	**6**	**7**	**8**	**9**
(1) Feminist Id.	3.98 (1.72)	–	–0.57^∗∗^	–0.34^∗∗^	0.52^∗∗^	–0.20^∗∗^	0.34^∗∗^	0.51^∗∗^	0.42^∗∗^	0.41^∗∗^
(2) HS	1.36 (1.17)		–	0.65^∗∗^	–0.25^∗∗^	0.50^∗∗^	–0.19^∗∗^	–0.42^∗∗^	–0.22^∗∗^	–0.30^∗∗^
(3) BS	1.11(0.91)			–	–0.06	0.61^∗∗^	–0.01	−0.17^∗^	–0.10	−0.15^∗^
(4) Egalitarian Mot.	4.73 (1.49)				–	0.18^∗^	0.60^∗∗^	0.48^∗∗^	0.42^∗∗^	0.22^∗∗^
(5) Paternalistic Mot.	2.83 (1.46)					–	0.14	–0.04	–0.14	–0.21^∗∗^
(6) Assertive Confr.	3.97 (1.50)						–	0.35^∗∗^	0.28^∗∗^	0.16^∗^
(7) Support CCA	4.98 (1.34)							–	0.37^∗∗^	0.25^∗∗^
(8) Interest “MxEq Mov.”	6.86 (2.72)								–	0.48^∗∗^
(9) Engagement “MxEq Mov.”	0.63 (0.68)									–

*Id., Identification; HS, Hostile Sexism; BS, Benevolent Sexism; Mot., Motivation; Confr., Confrontation; CCA, Collective Actions; MxEq Mov., Men for equity movement. The items in all scales ranged from 1 to 7, with the exception of Interest “MxEq Mov.” that ranged from 0 to 10 and Engagement “MxEq Mov.” that ranged from 0 to 2. ^∗^*p* < 0.05, ^∗∗^*p* < 0.01.*

#### Motivational Processes Underlying Sexism Confrontation in Men

We conducted the same two mediational models as in Study 1 to test Hypotheses 1a and 1b. Benevolent and hostile sexism correlated (*r* = 0.65, *p* < 0.01), so we included hostile sexism as a covariate. The total effect was significant in both models (feminist identification: *b* = 0.30, 95% CI [0.18,0.42]; benevolent sexism: *b* = 0.38, 95% CI [0.08,0.69]), as well as the indirect effects on assertive confrontation of feminist identification via egalitarian motivation (*b* = 0.26, 95% CI [0.20,0.34]) and of benevolent sexism via paternalistic motivation (*b* = 0.24, 95% CI [0.11,0.42]). The direct effects were non-significant when the mediators were included in the models (feminist identification: *b* = 0.04, 95% CI [−0.08,0.15]; benevolent sexism: *b* = 0.14, 95% CI [−0.19,0.47]; see Figure 1 and Table 4). Thus, Hypotheses 1a and 1b were supported.

#### Men’s Support for Collective Action, Interest in Men for Equity Movement, and Engagement in the Movement

To test Hypotheses 2a and 2b, we analyzed the role of feminist identification and benevolent sexism as predictors of collective action intentions in support of women’s rights, mediated by egalitarian and paternalistic motivations. On the one hand, the total effect of feminist identification on collective action intentions was significant (*b* = 0.39, 95% CI [0.29,0.48]). As expected, there was an indirect effect of feminist identification on collective action intentions through egalitarian motivations (*b* = 0.13, 95% CI [0.06,0.21]). The direct effect of feminist identification remained significant (*b* = 0.26, 95% CI [0.15,0.36]). On the other hand, the total effect of benevolent sexism on collective action intentions was non-significant (*b* = 0.23, 95% CI [−0.02,0.49]). However, the indirect effect via paternalistic motivation was unexpectedly significant (*b* = 0.13, 95% CI [0.01,0.26]). The direct effect was non-significant (*b* = 0.11, 95% CI [−0.17,0.39]). Thus, Hypothesis 2a was confirmed with our data, but Hypothesis 2b was rejected.

We conducted the same analyses on men’s interest in knowing more about the Men for Equity movement to test Hypotheses 3a and 3b. The total effect of feminist identification was significant (*b* = 0.64, 95% CI [0.43,0.86]), as well as the indirect effect via egalitarian motivation (*b* = 0.24, 95% CI [0.09,0.42]). Direct effect remained significant when we included the mediator in the model (*b* = 0.40, 95% CI [0.16,0.64]). However, interest in the Men for Equity movement was not predicted by benevolent sexism (total effect: *b* = 0.15, 95% CI [−0.45,0.75]; indirect effect via paternalistic motivation: *b* = −0.12 95% CI [−0.40,0.14]; direct effect: *b* = 0.27, 95% CI [−0.40,0.93]). Thus, Hypothesis 3a and 3b were supported.

Finally, we conducted two more mediational models to test the motivational processes with the true intentions to engage in Men for Equity groups (Hypotheses 4a and 4b). In this case, the total effect of feminist identification on intentions to engage in the movement was significant (*b* = 0.16, 95% CI [0.10,0.22]), although the indirect effect through egalitarian motivation was not (*b* = 0.00, 95% CI [−0.03,0.04]). Direct effect remained significant when the mediator was included in the model (*b* = −0.17, 95% CI [0.11,0.24]). Engagement in the activist movement was not predicted by benevolent sexism as expected (total effect: *b* = 0.07, 95% CI [−0.07,0.22]; indirect effect via paternalistic motivation: *b* = −0.04, 95% CI [−0.13,0.03]; direct effect: *b* = 0.12, 95% CI [−0.05,0.28]). Thus, Hypothesis 4a was partially supported because feminist identification predicted engagement in the movement directly but not indirectly via egalitarian motivation, whereas Hypothesis 4b was supported.

### Discussion

The results of Study 2 supported most of our hypotheses, replicating the pattern found in Study 1 for the paternalistic path and giving empirical support to the egalitarian path. In this case, with a larger sample, we improved the statistical powers for both paths to a value higher than 0.75, according to the Monte Carlo simulation approach. In addition, our results also extended previous findings to actions more related to social change and actual behavior. The paternalistic path also unexpectedly predicted men’s support for collective action. However, only the feminist path predicted involvement in actions that imply reconsidering their role in gender inequality directly. These results suggest that paternalistic motivations might sometimes promote men to at least declare having intentions to participate in collective action for women’s rights (but see Radke et al., 2018), however, they do not motivate either their interest in or commitment to men’s activism against gender inequality. Overall, the results of Study 2 support our proposed two motivational paths to sexism confrontation and provide preliminary evidence suggesting that only the feminist path can promote social change. However, some of the results were unexpected and the statistical power did not reach the conventional 0.80 value (Fritz and MacKinnon, 2007). To overcome these limitations, we conducted Study 3 as a close conceptual replication; we also preregistered it in the Open Science Framework platform.

## Study 3

In this study, we expected to replicate the two motivational processes underlying sexism confrontation in men, as well as collective action intentions, interest in the Men for Equity movement, and willingness to engage in this movement. As in previous studies, we hypothesized that feminist identification predicts assertive confrontation via egalitarian motivation (Hypothesis 1a), whereas benevolent sexism predicts assertive confrontation via paternalistic motivation (Hypothesis 1b). To replicate Study 2, we hypothesized that feminist identification predicts both support for collective action (Hypothesis 2a), interest in the Men for Equity movement (Hypothesis 3a), and actual engagement in this movement (Hypothesis 4a) via egalitarian motivation, whereas benevolent sexism and paternalistic motivation are not related to these outcomes (Hypotheses 2b, 3b and 4b). The study hypotheses and methods were preregistered and are available at https://osf.io/euyfx.

### Method

#### Participants

A total of 253 men completed the survey. We excluded a multivariate outlier so the final sample comprised 252 men. Participants’ ages ranged from 18 to 46 years old with a mean of 22.59 years (*SD* = 4.34). Of the sample, 84.9% were students from a university in southern Spain, whereas 15.1% were workers or were applying for a job in public administration; 96% were Spanish citizens. Based on the data from our previous studies, we calculated *a priori* the sample needed to obtain a power of 0.80. We use two different approaches: Monte Carlo simulation to estimate statistical power in mediation models (Schoemann et al., 2017) and the recommendations of Fritz and MacKinnon (2007) regarding a bias-corrected bootstrapping approach in mediation analyses. Both approaches indicated that a sample size of 250 was adequate to raise a power of 0.80.

#### Measures

We mainly used the same measures as in Study 2 (unless otherwise specified), reducing the number of items when possible to shorten the questionnaire (the questionnaire is available at https://osf.io/xcae7/). The response scales were the same as in the previous studies.

##### Demographics

We recorded participants’ ages, nationality, and career areas.

##### Feminist identification

We used the same seven items as in Studies 1 and 2 (α = 0.95).

##### Hostile and benevolent sexism

We used the short 12-item version of the Ambivalent Sexism Inventory (Rollero et al., 2014). Six items were used to measure hostile sexism (SHα = 0.86) and six items to measure benevolent sexism (SBα = 0.74).

##### Sexist situation

We only included one sexist situation using two comic vignettes developed for another set of studies (Estevan-Reina et al., unpublished). In the first vignette, a woman asked some men standing at the entrance of a club for a lighter. In the second vignette, one of the men said, “Of course I will lend it to you, pretty girl, but if in return you come to sleep with me tonight, because I don’t want to sleep alone.”

##### Intentions of assertive confrontation

We used the same 12 items as in Study 2 (α = 0.97).

##### Motivations underlying assertive sexist confrontation

We used the same four items used in previous studies to measure paternalistic motivation (α = 0.76) and four of the eight items used in previous studies to measure egalitarian motivation (α = 0.94). Since in this study we have a sufficient sample size to carry out a confirmatory factor analysis (Byrne, 2001), we decided to verify with this technique that the items used to measure paternalistic motivation constitute an independent factor of benevolent sexism. To this aim we tested two models with AMOS 25.0. Model A included two latent variables (benevolent sexism and paternalistic motivation) and Model B one single-factor. The goodness of fit showed a better adjustment of Model A [χ*^2^* = 54.56, *df* = 26, *p* = 0.001; χ*^2^/df* = 2.10; *RMSEA* = 0.07 (*PCLOSE* = 0.11), *CFI* = 0.97, *NFI* = 0.94] compared to Model B [χ*^2^* = 93.38, *df* = 27, *p* ≤ 0.001; χ*^2^/df* = 3.46; *RMSEA* = 0.10 (*PCLOSE* > 0.001), CFI = 0.92, NFI = 0.90]. A chi square different test (Δχ^2^ = 38.82, *p* = 0.05) and the comparison between the models using AIC (Akaike, 1974) (AIC Model A = 132.56; AIC Model B = 169.38), confirmed that Model A had a better adjustment.

##### Intentions of supporting collective actions

We used the four items developed by Radke et al. (2018) to measure willingness to engage in feminist actions (e.g., “I will protest against sexism”; α = 0.87) and four more items (pretested by Estevan-Reina et al., unpublished) that represented actions aimed to question male privilege and to support redistribution of power and responsibilities between women and men (e.g., “Participate in activities in which male privileges in current society are questioned”). The response scale varied from 1 (very unlikely) to 7 (very likely).

##### Interest in the men for equity movement

We used the same single item as in Study 2.

##### Intentions to engage in men for equity groups

As in Study 2, this measure was composed of two items. The results showed that 25.9% of the participants were open to receiving more information about the Men for Equity movement in Spain, but only 62.5% of those who had shown interest in the movement agreed to provide their e-mail addresses to be directly contacted by the organization (16.6% of the total participants). With the information obtained for both items, we created a new variable ranging from 0 (*no engagement in these groups*) to 2 (*maximum engagement in these groups*).

#### Procedure

We approached men at some university libraries and asked for their collaboration to complete a 10-min paper-and-pencil questionnaire. After completion, we rewarded participants with a chocolate bar.

### Results

#### Analytical Strategy

We conducted correlational analyses to test the relationships among our variables (see Table 3). Then we ran mediational analyses with the macro PROCESS to confirm the two motivational processes underlying sexism confrontation among men (Hypotheses 1a and 1b), as well as to examine the role of feminism and paternalism to motivate men in fighting gender inequality through other types of behaviors such as supporting collective action (Hypotheses 2a and 2b), showing interest in the Men for Equity movement (Hypotheses 3a and 3b), or actively engaging in it (Hypotheses 4a and 4b).

**TABLE 3:** Means, standard deviations, and correlations for Study 3.

	**Mean (*SD*)**	**1**	**2**	**3**	**4**	**5**	**6**	**7**	**8**	**9**
(1) Feminist Id.	4.55 (1.69)	–	–0.62^∗∗^	–0.32^∗∗^	0.69^∗∗^	–0.18^∗∗^	0.47^∗∗^	0.72^∗∗^	0.45^∗∗^	0.39^∗∗^
(2) HS	1.06 (1.00)		–	0.55^∗∗^	–0.52^∗∗^	0.28^∗∗^	–0.29^∗∗^	–0.52^∗∗^	–0.24^∗∗^	–0.29^∗∗^
(3) BS	1.25 (0.97)			–	–0.19^∗∗^	0.48^∗∗^	–0.06	–0.22^∗∗^	–0.12	–0.21^∗∗^
(4) Egalitarian Mot.	5.21 (1.81)				–	0.14^∗^	0.70^∗∗^	0.77^∗∗^	0.44^∗∗^	0.34^∗∗^
(5) Paternalistic Mot.	3.28 (1.46)					–	0.25^∗∗^	–0.05	–0.02	−0.14^∗^
(6) Assertive Confr.	4.73 (1.69)						–	0.63^∗∗^	0.37^∗∗^	0.28^∗∗^
(7) Support CCA	4.47 (1.39)							–	0.53^∗∗^	0.44^∗∗^
(8) Interest “MxEq Mov.”	5.51 (3.50)								–	0.47^∗∗^
(9) Engagement “MxEq Mov.”	0.42 (0.75)									–

*Id., Identification; HS, Hostile Sexism; BS, Benevolent Sexism; Mot., Motivation; Confr., Confrontation; CCA, Collective Actions; MxEq Mov., Men for equity movement. The items in all scales ranged from 1 to 7, with the exception of Interest “MxEq Mov.” that ranged from 0 to 10 and Engagement “MxEq Mov.” that ranged from 0 to 2. ^∗^*p* < 0.05, ^∗∗^*p* < 0.01.*

#### Motivational Processes Underlying Sexism Confrontation in Men

We conducted the same two simple mediational models as in previous studies (see Figure 1 and Table 4). Regarding the feminist path, the total effect of feminist identification on men’s future intentions to confront sexism was significant (*b* = 0.47, 95% CI [0.36,0.58]) as well as the indirect effect via egalitarian motivation (*b* = 0.49, 95% CI [0.39,0.59]). The direct effect was not significant when the egalitarian motivation was included as mediator (*b* = −0.01, 95% CI [−0.14,0.11]). Thus, Hypothesis 1a was supported.

**TABLE 4:** Summary of total, direct and indirect effect of feminist identification and benevolent sexism on men’s future intentions to confront Studies 1-3 and pooled analyses.

**Relationship between Feminist Identification and men’s future intentions to confront via egalitarian motivation**																

	**Study 1 (*N* = 144)**				**Study 2 (*N* = 196)**				**Study 3 (*N* = 252)**				**Pool analyses (*N* = 592)**			
				
	**Effect**	***SE***	**LLCI**	**ULCI**	**Effect**	***SE***	**LLCI**	**ULCI**	**Effect**	***SE***	**LLCI**	**ULCI**	**Effect**	***SE***	**LLCI**	**ULCI**
Total effect	0.20	0.08	0.04	0.35	0.30	0.06	0.18	0.42	0.47	0.06	0.36	0.58	0.37	0.04	0.30	0.45
Direct Effect	0.12	0.09	–0.06	0.30	0.04	0.06	–0.08	0.15	–0.01	0.06	–0.14	0.11	0.11	0.04	0.03	0.19
Indirect Effect	0.07	0.07	–0.01	0.19	0.26	0.04	0.20	0.34	0.49	0.05	0.39	0.59	0.27	0.03	0.21	0.33

**Relationship between Benevolent Sexism and men’s future intentions to confront via paternalistic motivation**																

	**Study 1 (*N* = 144)**				**Study 2 (*N* = 196)**				**Study 3 (*N* = 252)**				**Pool analyses (*N* = 592)**			
				
	**Effect**	***SE***	***LLCI***	***ULCI***	**Effect**	***SE***	***LLCI***	***ULCI***	**Effect**	***SE***	***LLCI***	***ULCI***	**Effect**	***SE***	***LLCI***	

Total effect	0.17	0.19	–0.21	0.55	0.38	0.15	0.08	0.84	0.25	0.12	0.00	0.50	0.32	0.09	0.14	0.49
Direct effect	–0.05	0.21	–0.46	0.37	0.14	0.17	–0.19	0.47	–0.04	0.13	–0.30	0.21	0.05	0.09	–0.13	0.24
Indirect effect	0.22	0.12	0.01	0.47	0.24	0.08	0.11	0.42	0.29	0.08	0.16	0.47	0.27	0.05	0.18	0.38

Benevolent and hostile sexism correlated (*r* = 0.55, *p* < 0.01) so we included hostile sexism as a covariate. Regarding the paternalistic path, the total effect of benevolent sexism on men’s future intentions to confront was significant (*b* = 0.25, 95% CI [0.00,0.50]) as well as the indirect effect via paternalistic motivation: *b* = 0.29, 95% CI [0.16,0.47]). The direct effect was not significant (*b* = −0.04, 95% CI [−0.30,0.21]). Thus, Hypothesis 1b was also supported.

#### Men’s Support for Collective Action, Interest in the Men for Equity Movement, and Engagement in That Movement

We conducted six more mediational analyses including men’s support for collective action, interest in the Men for Equity movement, and intentions to engage in this movement as outcomes. Regarding the feminist path, the total effects of feminist identification on the three outcome variables were significant (support for collective actions: *b* = 0.60, 95% CI [0.53,0.67]; interest for the Men for Equity movement: *b* = 0.94, 95% CI [0.71,1.18]; intentions to engage in the movement: *b* = 0.18, 95% CI [0.12,0.23]), as well as the indirect effects via egalitarian motivation (support for collective action: *b* = 0.29, 95% CI [0.23,0.36]; interest in the Men for Equity movement: *b* = 0.36, 95% CI [0.09,0.62]; engagement in the movement: *b* = 0.06, 95% CI [0.02,0.11]). The direct effects remained significant in all cases (support for collective action*: b* = 0.31, 95% CI [0.23,0.40]; interest in the Men for Equity movement: *b* = 0.58, 95% CI [0.27,0.90]; engagement in the movement:0.13, 95% CI [0.05,0.20]). Thus, Hypothesis 2a, 3a, and 4a were supported.

Further, as we expected, the total effects of benevolent sexism on the three outcome variables were not significant (support for collective actions: *b* = 0.15, 95% CI [−0.03,0.33]; interest in the Men for Equity movement: *b* = 0.06, 95% CI [−0.46,0.59]; engagement in the movement: *b* = −0.06, 95% CI [−0.17,0.05]), and neither were the indirect effects via paternalistic motivation (support for collective actions: *b* = 0.06, 95% CI [−0.02,0.15]; men’s interest in the Men for Equity movement: *b* = −0.08, 95% CI [−0.17,0.33]; engagement in the movement: −0.02, 95% CI [−0.07,0.03]) nor the direct effects (support for collective actions: *b* = 0.09, 95% CI [−0.11,0.29]; men’s interest in the Men for Equity movement: *b* = −0.02, 95% CI [−0.59,0.56]; engagement in the movement: −0.04, 95% CI [−0.17,0.08]). Thus, Hypotheses 2b, 3b, and 4b were also supported.

### Discussion

Results of Study 3 confirmed the existence of two different routes that lead men to confront sexism, a feminist path and an egalitarian path, but in this case using a larger sample that guaranteed sufficient statistical power and a preregistered conceptual replication of Study 2’s findings. In Study 3, we also confirmed that although the paternalistic route predicts sexism confrontation among men it does not relate to the involvement in other types of outcomes against inequality (such as supporting collective action, interest in the Men for Equity movement, or engagement in this movement). Thus, in these last actions, only the feminist path played a significant role.

## Pooled Analyses

Whereas the results of Studies 2 and 3 are highly symmetrical, there were some discrepancies between the results from these two studies and Study 1. For this reason, to test our hypotheses considering all the evidence gathered and to seek convergence between studies, we conducted integrative data analysis (Curran and Hussong, 2009). This procedure allowed us to increase statistical power and sample heterogeneity. First we pooled the samples from the three studies into a single analysis to confirm both paths underlying sexism confrontation. Total sample included 592 participants (*N*1 = 144; *N*2 = 196; *N*3 = 252). Then, we pooled the samples of Studies 2 and 3 to confirm the relevance of the feminist path in predicting other types of actions beyond sexism confrontation (collective action, interest and engagement in an activist movement). The total sample included 448 participants (*N*2 = 196; *N*3 = 252).

### Motivational Processes Underlying Sexism Confrontation in Men (Pooled Analysis of Studies 1–3)

Integrative data analysis confirmed the paternalist and egalitarian paths underlying men’s confrontation of sexism. The total effect of feminist identification on men’s intentions to confront was significant (*b* = 0.37, 95% CI [0.30,0.45]), as well as the indirect effect via egalitarian motivation (*b* = 0.27, 95% CI [0.21,0.33]). The direct effect remained significant when egalitarian motivation was controlled for (*b* = 0.11, 95% CI [0.03,0.19]). Further, the total effect of benevolent sexism on men’s intentions to confront was significant (*b* = 0.32, 95% CI [0.14,0.49]), as well as the indirect effect via paternalistic motivation (*b* = 0.27, 95% CI [0.18,0.38]). The direct effect was non-significant (*b* = 0.05, 95% CI [−0.13,0.24]; see Figure 1 and Table 4).

**FIGURE 2:**
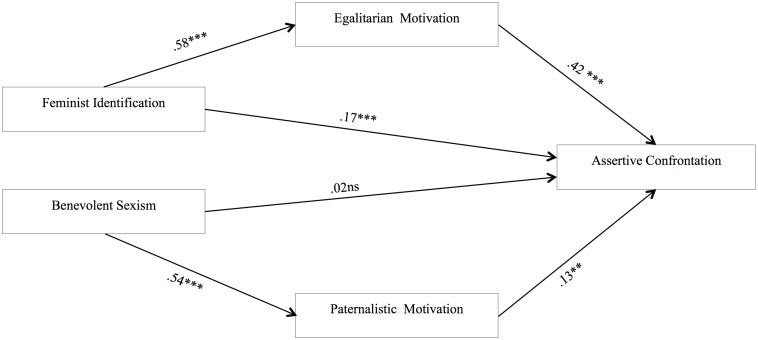
Path analysis with pooled data of Studies 1–3 to test simultaneously the two motivational processes (feminist and paternalistic path) underlying men’s future intensions to confront. ^∗^*p* < 0.05, ^∗∗^*p* < 0.01, ^∗∗∗^*p* < 0.001.

Moreover, using the pooled data we conducted a path analyses (Byrne, 2001) with AMOS 25.0 to confirm that both processes occur independently when controlling for the other simultaneously (Model A, see Figure 2). The overall fit of Model A was excellent, the chi square was non-significant, the RMSEA is above the 0.06 cut off, and the CFI and NFI values are below 0.95, χ^2^ = 3.04, df = 2, *p* = 0.22; χ^2^/df = 1.52; RMSEA = 0.03 (PCLOSE = 0.61), CFI = 0.99, NFI = 0.99. A modification of this model controlling for the effect of hostile sexism on paternalistic motivation (Model B) had also a good adjustment [χ^2^ = 17.52, df = 4, *p* = 0.002; χ^2^/df = 4.38; RMSEA = 0.08 (PCLOSE = 0.10), CFI = 0.99, NFI = 0.99] although the Akaike information criterion (AIC; Akaike, 1974) is larger in Model B (AIC = 51.52) than Model A (AIC = 29.04) which implies a better fit of Model A. Finally, given that this was a correlational study and it was not possible to be certain about the direction of causality between the variables, one new model was tested inverting the direction of the predictive relationships between feminism/sexism and motivations. Model C tested the hypothesis that egalitarian motivation influenced confrontation via feminist identification, whereas paternalistic motivation influenced confrontation via benevolent sexism. Model C presented inappropriate fit indexes, χ^2^ = 114.05, df = 2, *p* ≤ 0.001; χ^2^/df = 57.02; RMSEA = 0.310 (PCLOSE ≤ 0.001), CFI = 0.86, NFI = 0.86. A chi square different test, Δχ^2^ = 111.01, *p* < 0.001, showed that Model A had better goodness-of-fit indexes than Model C. Both models were also compared by using the Akaike information criterion. Model A showed a smaller AIC than Model C (AIC = 140.05), which also implies a better fit of the former.

### Men’s Support for Collective Action, Interest in the Men for Equity Movement, and Engagement in the Movement (Pooled Analysis of Studies 2–3)

We tested the predictive models on collective action intentions and interest and engagement in the Men for Equity movement with the pooled sample of Studies 2–3. The results revealed that total effects of feminist identification on the three outcome variables were significant (support for collective actions: *b* = 0.47, 95% CI [0.41,0.53]; interest in the Men for Equity movement: *b* = 0.74, 95% CI [0.58,0.91]; engagement in the movement: *b* = 0.15, 95% CI [0.12,0.19]). The indirect effects via egalitarian motivation were significant on support for collective action (*b* = 0.21, 95% CI [0.15,0.26]) and on interest in the Men for Equity movement (*b* = 0.30, 95% CI [0.15,0.47]), but not significant on intentions to engage in the movement (*b* = 0.02, 95% CI [−0.01,0.05]). The direct effects remained significant in all cases (men’s support for collective actions*: b* = 0.26, 95% CI [0.19,0.33]; interest in the Men for Equity movement: *b* = 0.44, 95% CI [0.23,0.65]; engagement in the movement: *b* = 0.14, 95% CI [0.09,0.19]). The results showed that feminist identification directly predicted engagement in the Men for Equity movement, as well as support for collective action and interest in the Men for Equity movement (both directly and indirectly through egalitarian motivation).

Regarding the paternalistic path, the total effects of benevolent sexism were non-significant in any of the three outcome variables (support for collective actions: *b* = 0.09, 95% CI [−0.06,0.25]; interest in the Men for Equity movement: *b* = −0.08, 95% CI [−0.48,0.32]; engagement in the movement: *b* = −0.04, 95% CI [−0.13,0.04]). The indirect effects via paternalistic motivation were non-significant on support for collective action (*b* = 0.06, 95% CI [−0.02,0.13]) and men’s interest in the Men for Equity movement (*b* = −0.04, 95% CI [−0.25,0.15]), but it was significant although negative on engagement in the movement: (*b* = −0.04, 95% CI [−0.09,−0.00]). The direct effects were non-significant (support for collective actions*: b* = 0.04, 95% CI [−0.13,0.20]; men’s interest in the Men for Equity movement: *b* = −0.04, 95% CI [−0.48,0.41]; engagement in the movement: *b* = 0.00, 95% CI [−0.10,0.10]). These results showed that benevolent sexism not only did not predict collective actions, but it also inhibited engagement in the Men for Equity movement via paternalistic motivation.

## General Discussion

In recent years, thanks mainly to the Me Too movement, awareness about sexual harassment has grown worldwide. Although this and previous similar movements have been led by women, more and more consciousness is arising about the importance of encouraging men to join the fight against sexual harassment and gender discrimination more generally. But the implication of men in this endeavor merits some detailed analysis. Our studies have shown that when men face sexism, they can be motivated to confront it by different reasons, but they do not have the same impact in promoting other actions in fighting gender inequality. The results of this set of studies support the existence of two paths that lead men to confront sexism (specifically episodes of street sexual harassment): one of them mostly based on feminism and the other mostly based on paternalism. Feminist identification predicts sexism confrontation directly and indirectly via egalitarian motivation, whereas benevolent sexism predicts sexism confrontation indirectly via paternalistic motivation. However, only the feminist path consistently predicts men’s support for collective action, interest in the Men for Equity movement, and actual engagement in that movement. Thus, these results confirm the boundaries of paternalism compared with feminism in promoting social change.

On the one hand, our results show that egalitarian motivation plays an important role in explaining sexism confrontation, supporting collective action and interest in the Men for Equity movement. Confrontation is an individual action that takes place in the interpersonal context, and it refers to challenging a perceived injustice in how someone is treated due to his or her social background; whereas men’s support for feminist actions and men’s interest in the Men for Equity movement are actions that question the *status quo* to a greater extent. However, despite the differentiated nature of these actions, varying from the interpersonal to the intergroup level (see Stroebe et al., 2015, for a reflection on collective and individual actions), our results show that they share the same underlying motivational mechanism. This may be due to the fact that confronting sexism, being an individual action, could be understood as a collective strategy in coping with daily prejudice that involves two processes: (a) not only emphasizing the inappropriateness of a comment in an interpersonal setting, but (b) improving the situation of women as a group (Becker et al., 2015). However, egalitarian motivation did not play such a relevant role in predicting men’s engagement with the Men for Equity movement. The difference between intention and behavior (Sheeran and Webb, 2016) might account for this fact. Whereas motivations might be a closer predictor of behavioral intentions, other factors might explain the link to actual behavior. Besides, in the case of men as an advantaged group, the content of the feminist identity should also imply a deeper reflection on their own privileged position and a commitment to changing it (in-group focused), and this process seems different from the egalitarian motivation that can emerge in a specific situation (out-group focused). That is, the egalitarian motivation path is based on a reflection about intergroup relations and willingness to improve the out-group’s disadvantaged position, whereas deciding to actively participate in a profeminist men’s movement implies rethinking men’s identity and privileges. Although both aspects can be needed to balance the in-group–out-group disadvantage, they might be explained by different underlying motivational processes.

On the other hand, our work also highlights that the paternalistic motivation is the mechanism that explains the relationship between benevolent sexism and confrontation, and we suggest that it may be an important factor in order to understand the relationship found in some previous work between benevolent sexism and actions related to protecting women (e.g., dependency-oriented helping, Shnabel et al., 2016; protective collective action, Radke et al., 2018). Besides, the fact that the paternalistic path does not predict either collective action or interest in male activism confirms the limits of paternalism in promoting social change. In fact, pooled analyses showed that benevolent sexism via paternalistic motivation not only does not increase men’s engagement with the Men for Equity movement, but it actually inhibits their participation. In some sense, these results are consistent with the idea that certain “egalitarian” narratives built by men do not promote social change unless these discourses are translated into practice (Lamont, 2015).

### Limitations and Future Research

We are aware that this work also has some limitations. Our samples are limited to Spanish college students. In recent years, gender issues have become an important political agenda topic in Spain (Grodira et al., 2018), so despite the feminist stigma (Anderson, 2009; Anastosopoulos and Desmarais, 2015), feminist identification is rising in Spain (Álvarez, 2018; Centro de Investigaciones Sociológicas [Sociological Research Center], 2018). Thus, it would be important to replicate these results in other countries, as well as to study these processes with the general population to see if they can be generalized. In the same way, the sexist comments that we used in our scenarios described sexist situations based on sexual objectification of women or undesired sexual attention that happens in a leisure context. We chose this context because it is closer to college students’ real experiences and because the previous literature showed that it is the type of sexist incident that only women suffer (compared with men) (Swim et al., 2001). Although analyzing the motivational process that leads men to face sexual harassment is important, future researchers should check whether the proposed motivational processes are also valid for explaining the assertive confrontation of other types of (non-sexual) sexist incidents. Another limitation of our work is the lack of experimental evidence to establish causality in the relationship among our variables. In Study 2, we tried to overcome this limitation, but we did not succeed. Perhaps future researchers should focus on manipulating the underlying motivations instead of attitudinal variables that can be more difficult to influence. Another factor that could be further explored is the moderating role of the severity of the sexist event. Previous research found that the likelihood to intervene in more severe sexist incidents was higher than in less severe ones (Chabot et al., 2009). Manipulating the severity of sexist situations may be a way to activate paternalistic motivation because the duty to protect women when they are at risk is a central aspect of traditional masculinity (Thompson and Bennett, 2015). Further, it should be interesting to test our hypotheses using community samples because older and less educated populations usually endorse more traditional gender attitudes than college students. Concerning the measures used in our studies, it is important to notice that some of them were not previously subjected to an exhaustive validation process. So to guarantee their ecological and construct validity more research is needed. Finally, we cannot ignore that intentions are often only weakly predictive of actual behavior and particularly inconsistent when scenarios are threatening (for a related discussion in the context of real versus imagined gender harassment see Woodzicka and LaFrance, 2001). Future research should address this issue testing our predictions in a real confrontation context.

Overall, this work is a first step to understanding the ideological and motivational factors underlying men’s confrontations of sexism. Future research should explore the implications for women (as targets of discrimination) of men’s confrontations of sexism guided by paternalistic or feminist paths, as well as whether these motivations affect women’s perception of confronters as allies. Further, future research should examine if paternalistic confrontation actually perpetuates inequality. Paternalistic motivation is a way in which the masculine belief (Saucier et al., 2016) in the “duty to protect women” (Good et al., 2016) is expressed. Thus, confrontation motivated by paternalistic reasons may allow men to project the image of being non-sexist, at the same time that it reinforces their masculinity, allowing them to appear as chivalrous men. In a similar way, confronting sexism based on paternalistic reasons can allow men to use “egalitarian” arguments as a way of constructing the understanding of themselves as progressive, caring, and respectful of women, in contrast to the majority of men (Lamont, 2015), at the same time that they are reinforcing traditional gender roles. In addition, future researchers should analyze the role of reflection about male privilege as a mediator between feminist identification and out-group-focused actions (such as engagement in the Men for Equity movement), as well as the role that it can play in men becoming genuine allies of women in fighting gender inequality.

## Conclusion

These three studies have added some relevant knowledge to the current literature on the role played by men in confronting sexism and its implication in different collective actions in favor of gender equality. The results confirm the importance of men’s feminist identification in promoting social change through confronting sexism, in line with findings in the literature on collective action (Wiley et al., 2012; Radke et al., 2018). We have identified two motivational paths in men fighting gender inequality. Our results confirm that both feminist identification via egalitarian motivation and benevolent sexism via paternalistic motivation may help to explain men’s attempts to confront sexism. In addition, this work highlights the potentialities of feminism compared to paternalism to promote social change. Taking these results into account, considering these different motivations underlying actions against inequality may be helpful to understanding when men could be true allies of women in promoting social change, or on the contrary when they could contribute to perpetuating the *status quo*. From an applied point of view, we emphasize that the social interventions aimed at combating gender inequality should be focused on the development of feminist identity in men, and on the promotion of egalitarian motivations instead of paternalistic ones.

## Data Availability Statement

The datasets generated for pooled analyses can be found in the Open Science Framework: https://osf.io/fjbk4/. The raw data supporting the conclusions of Studies 1–3 will be made available by the authors, without undue reservation, to any qualified researcher.

## Ethics Statement

The studies involving human participants were reviewed and approved by the Comité de Ética de la Universidad de Granada. Written informed consent for participation was not required for this study in accordance with the national legislation and the institutional requirements.

## Author Contributions

All authors have made a significant contributions to the present work designing the studies and analyzing data. LE-R drafted the manuscript. SL and JM provided feedback in the manuscript revision.

## Conflict of Interest

The authors declare that the research was conducted in the absence of any commercial or financial relationships that could be construed as a potential conflict of interest.
